# Decoding Autoimmunity: Insights Into Neuromyelitis Optica and Its Relationship With Other Autoimmune Neurological Disorders

**DOI:** 10.7759/cureus.82062

**Published:** 2025-04-11

**Authors:** Tabish W Siddiqui, Raqshan W Siddiqui, Shiza W Siddiqui, Sohaila Fatima, Hiba R Babu, Uvashree Shrinivas, Leah L Dias, Jefina Olive

**Affiliations:** 1 Department of Medicine, RAK Medical and Health Sciences University, Ras al Khaimah, ARE; 2 Department of Medicine, RAK Medical and Health Sciences University, Ras Al Khaimah, ARE; 3 Department of Internal Medicine, King Khalid University Hospital, Abha, SAU; 4 Department of Pathology, King Khalid University, Abha, SAU

**Keywords:** autoantibodies, autoimmune diseases, biomarkers, multiple sclerosis, neuromyelitis optica

## Abstract

Neuromyelitis optica (NMO) is a rare but debilitating autoimmune condition characterized by severe attacks of optic neuritis and transverse myelitis, often resulting in significant neurological disability. Autoantibodies targeting aquaporin-4 (AQP4) and myelin oligodendrocyte glycoprotein (MOG) play critical roles in disease pathogenesis, and therapeutic strategies encompassing immunosuppressive therapies and emerging biologics are employed to manage disease activity and prevent relapse. While studies suggest a potential overlap between NMO and other autoimmune neurological conditions, research in this area remains limited. This review explores the commonalities and distinctions between NMO and related autoimmune neurological disorders, hypothesizing that shared autoantibody mechanisms and clinical features may refine diagnostic criteria and therapeutic interventions. Additionally, it addresses tailored management approaches for specific clinical features of NMO and its overlaps. The paper also explores current research on biomarkers and novel treatment modalities, highlighting persistent knowledge gaps, such as understanding the immune mechanisms behind NMO and predicting individual responses to therapies. The review underscores the necessity for collaborative research efforts to improve diagnostic accuracy and therapeutic efficacy. Ultimately, these efforts will enhance personalized care strategies and optimize outcomes and quality of life for patients with NMO and related autoimmune neurological disorders.

## Introduction and background

Damage to the myelin sheath, which insulates nerve axons, leads to central nervous system (CNS) demyelination, resulting in impaired action potential conduction, reduced neuronal signaling, and potentially neuronal loss [1]. While the etiology of primary demyelination often remains elusive, it is frequently associated with autoimmune processes, sometimes triggered by preceding viral infections. Other causes of demyelination can include metabolic disorders, ischemia, or inherited conditions [1].

The immune system's precise regulation is critical in distinguishing self from non-self, maintaining tolerance, and preventing autoimmunity. Central to this regulation are T lymphocytes, including regulatory T cells (Tregs) and effector T cells (Teffs) [2]. Tregs, characterized by the expression of the transcription factor FoxP3, are a subset of T cells that suppress immune responses, preventing the activation of self-reactive lymphocytes [2]. In contrast, Teffs, encompassing various subtypes like T helper 1 (Th1), Th2, and Th17 cells, promote inflammation by releasing cytokines and directly interacting with target cells [2]. B lymphocytes (B cells) play a crucial role in humoral immunity by producing antibodies, including autoantibodies in autoimmune diseases [2]. These cells can be activated by T cells and differentiate into plasma cells to produce and secrete large amounts of antibodies [2]. B cells also function as antigen-presenting cells (APCs), further promoting T cell activation [2].

In autoimmune disorders, this carefully orchestrated system malfunctions, leading to a breakdown in self-tolerance and immune-mediated damage of neural tissues [1]. There is often a numerical and functional deficiency of Tregs, leading to inadequate suppression of autoreactive lymphocytes [2]. Additionally, Teffs, particularly Th17 cells, are often hyperactivated, releasing pro-inflammatory cytokines like IL-17 that contribute to CNS inflammation [2]. This pro-inflammatory milieu further promotes the activation and differentiation of B cells into antibody-secreting plasma cells, culminating in the production of pathogenic autoantibodies [1,2].

Neuromyelitis optica (NMO), also known as Devic’s disease, is an autoimmune inflammatory demyelinating disease affecting the CNS, preferentially targeting the spinal cord, optic nerves, and certain brain regions [3]. The hallmark features of NMO are optic neuritis (ON) and longitudinally extensive transverse myelitis (LETM), often presenting with a relapsing-remitting course [2]. NMO is characterized by the presence of autoantibodies, primarily against aquaporin-4 (AQP4) and myelin oligodendrocyte glycoprotein (MOG) [1]. These antibodies play a critical role in disease pathogenesis by targeting astrocytes and oligodendrocytes, respectively [1].

Multiple sclerosis (MS) is another chronic inflammatory neurological condition characterized by inflammation, demyelination, gliosis, and neuronal death, distinguished by the presence of disseminated white matter lesions or plaques throughout the CNS [4]. While both NMO and MS are autoimmune-mediated demyelinating disorders, they differ in their immunopathogenesis, lesion distribution, and clinical course [1,4]. The immunopathology of MS involves the activation of autoreactive T cells that infiltrate the CNS, leading to inflammation and demyelination [4]. Compared to MS, NMO is characterized by more severe and relapsing ON and LETM [3]. Differentiating NMO from other autoimmune conditions can be challenging.

Autoimmune encephalitis (AIE) is an immune-mediated condition in which the host immune system targets self-antigens expressed in the CNS, causing brain inflammation. AIE is recognized as a frequent cause of acute noninfectious encephalitis [5]. Sjögren's syndrome, an autoimmune connective tissue disease affecting exocrine glands, also presents with neurological manifestations involving both the peripheral and CNS [6]. Systemic lupus erythematosus (SLE) is a multisystem autoimmune inflammatory disease with diverse clinical manifestations affecting nearly every organ in the body. Neurological complications in SLE include neuropsychiatric systemic lupus erythematosus (NPSLE) [7].

This review aims to compare NMO with MS, AIE, Sjögren's syndrome, and SLE, focusing on their pathophysiology, clinical manifestations, diagnostic challenges, and treatment approaches. The goal is to highlight differentiating features of NMO while acknowledging the overlap with other conditions, as well as address knowledge gaps to propose potential avenues for personalized treatment strategies, with an emphasis on CNS involvement and the role of specific autoantibodies.

To ensure the review is based on relevant and current information, a comprehensive literature search was conducted across multiple databases, including PubMed, Google Scholar, and Scopus using the following keywords: "neuromyelitis optica," "multiple sclerosis," "autoimmune encephalitis," "Sjögren's syndrome," "systemic lupus erythematosus," and "autoantibodies." Studies were selected based on their relevance to the key topics of pathophysiology, clinical manifestations, diagnostic approaches, and treatment strategies. Studies not directly related to these topics were excluded. While no formal risk of bias assessment was performed, studies were critically evaluated for methodological rigor and relevance to the review’s objectives.

## Review

Antibodies

Immune dysregulation and neurological injury result from a complex interplay between genetic predisposition and environmental stimuli in the pathogenesis of autoimmune disorders of the CNS [7]. In genetically predisposed individuals, environmental triggers like viral infections may initiate an autoimmune reaction [1].

A critical aspect of immune system function is its ability to distinguish self from non-self [1,2]. This distinction is essential to maintain immune tolerance and prevent autoimmune responses. Under normal circumstances, the immune system recognizes self-antigens, such as the body's own proteins and cells, as non-threatening, while non-self-antigens, such as pathogens, are targeted for destruction [1,2]. This process is regulated by a tightly controlled network of immune cells. One key player in immune regulation are Tregs, which suppress immune responses and prevent the activation of self-reactive lymphocytes through the transcription factor FoxP3 [2]. In contrast, effector Teffs, including Th1, Th2, and Th17 subsets, promote inflammation by releasing pro-inflammatory cytokines and interacting directly with target cells [2]. Additionally, B cells contribute to the immune response by producing antibodies, including autoantibodies, and act as APCs, further stimulating T cell activation [2].

In autoimmune disorders, this process becomes disrupted [1]. There is often a deficiency of Tregs, leading to inadequate suppression of self-reactive lymphocytes [1,2]. Simultaneously, Teffs, especially Th17 cells, are frequently hyperactivated and secrete inflammatory cytokines like IL-17, contributing to CNS inflammation [2]. In this altered immune landscape, immune effector cells - primarily T cells and B cells - become activated and produce autoantibodies. These antibodies, such as those targeting myelin in MS or AQP4 in NMO, bind to crucial CNS components, triggering immune-mediated damage and sparking an inflammatory cascade. This cascade promotes the infiltration of immune cells into the CNS, resulting in neural tissue damage and neurodegeneration following demyelination [4]. The result is a range of neurological symptoms [4].

The specific mechanisms underlying immune system disruption in autoimmune diseases are complex and multifactorial. One critical factor is deoxyribonucleic acid (DNA) damage, which, when accumulated, can impair DNA repair mechanisms and initiate autoimmune responses [8]. Evidence suggests that the accumulation of DNA damage activates the cyclic guanosine monophosphate-adenosine monophosphate (GMP-AMP) synthase (cGAS)-stimulator of interferon genes (STING) pathway, a key component of the innate immune response to cytosolic DNA [8]. Activation of this pathway leads to the production of type I interferons and other pro-inflammatory mediators, driving chronic inflammation and amplifying the autoimmune response [8]. Dysregulation of the cGAS-STING pathway has been implicated in several autoimmune diseases, including SLE and MS [8]. In the context of CNS autoimmune disorders, such as NMO and MS, activation of the cGAS-STING pathway exacerbates the inflammatory environment, contributing to the breakdown of immune tolerance and accelerating tissue damage [2,4,8].

**Figure 1:**
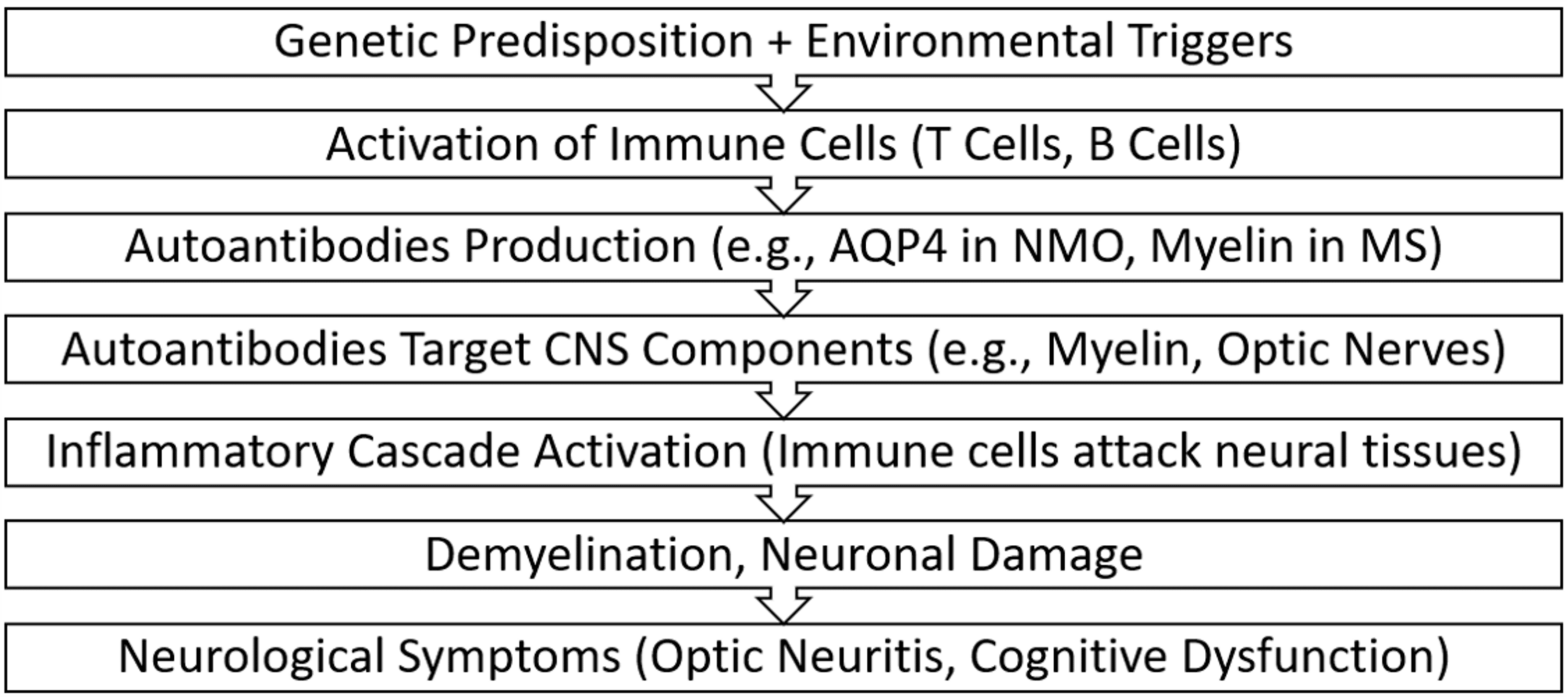
Overview of the immunopathology in autoimmune CNS diseases AQP4: aquaporin-4; NMO: neuromyelitis optica; MS: multiple sclerosis; CNS: central nervous system Image credits: Tabish Siddiqui

Figure 1 illustrates the immunopathological processes leading to CNS damage in autoimmune diseases, highlighting the cascade resulting in inflammation and tissue damage

Neuromyelitis Optica

NMO is characterized by the presence of serum IgG autoantibodies targeting AQP4, a water-channel protein predominantly found in astrocytes [1]. These antibodies play a central role in the pathogenesis of NMO by targeting astrocytes, leading to astrocyte dysfunction, complement activation, and subsequent demyelination and neuronal damage [3]. Clinically, AQP4-IgG seropositivity is strongly associated with severe, relapsing ON and LETM, resulting in significant visual and motor disability [3]. The detection of AQP4-IgG is crucial in distinguishing NMO from MS, as these antibodies are specific to NMO and not typically found in MS [1,4].

In a subset of patients, particularly those who are AQP4-negative, IgG antibodies targeting MOG are identified [1]. These patients often present with atypical NMO features, and MOG-IgG antibodies are more common in children and those with atypical NMO presentations [1]. Comprehensive antibody testing, particularly through cell-based assays, is essential for accurate diagnosis, as these assays provide the highest sensitivity for detecting both AQP4 and MOG antibodies [9,10].

Throughout the course of the disease, AQP4-IgG titers can fluctuate, particularly with disease activity or in response to immunosuppressive therapies. While AQP4-Abs often remain detectable during remission, their titer levels may decrease with treatment [10]. However, AQP4-Ab serostatus and titer levels do not reliably correlate with disease progression or response to immunotherapy, meaning that they are not reliable predictors of long-term outcomes or treatment efficacy [11,12]. Despite this, the presence of AQP4-IgG antibodies remains the key diagnostic criterion for NMO, guiding clinical decisions and treatment strategies, which may include immunosuppressants, B cell-depleting therapies, and complement inhibitors.

Multiple Sclerosis

MS is characterized by the presence of oligoclonal IgG bands in the cerebrospinal fluid (CSF), which reflect ongoing CNS inflammation and are considered a hallmark of the disease [13]. These oligoclonal bands are detected through isoelectric focusing and represent clonally expanded B cells [13]. While they are often used in diagnosing MS, oligoclonal bands are not exclusive to this disease and can also be present in other neuroinflammatory conditions and viral CNS infections [14,15]. As such, their presence, while supportive of an MS diagnosis, is insufficient on its own, and they need to be interpreted in conjunction with clinical symptoms and additional diagnostic tests.

In MS, the oligoclonal bands are thought to reflect B cell-mediated immune activation within the CNS, contributing to persistent inflammation and the development of demyelinating lesions [14]. Although these bands are a key diagnostic feature, their role in the progression of MS or in determining therapeutic responses remains unclear [14]. The presence of oligoclonal bands does not correlate strongly with clinical severity or long-term outcomes, and ongoing research aims to better understand their significance in disease course and treatment response [15].

Autoimmune Encephalitis

Autoimmune encephalitis is mediated by autoantibodies targeting neuronal cell-surface proteins and intracellular proteins. The clinical presentation of AIE is highly variable, depending on the specific autoantibodies involved [16]. Among the most common targets are N-methyl-D-aspartate (NMDA) receptors, leucine-rich glioma-inactivated 1 (LGI1) protein, contactin-associated protein-like 2 (Caspr2), alpha-amino-3-hydroxy-5-methyl-4-isoxazolepropionic acid (AMPA) receptors, and gamma-aminobutyric acid (GABA-B) receptors [16,17].

These antibodies lead to a direct autoimmune attack on neurons, causing a wide range of neurological symptoms. For example, anti-NMDAR encephalitis is often associated with psychiatric symptoms (such as psychosis and seizures), cognitive dysfunction, and movement disorders, while anti-LGI1 encephalitis typically presents with faciobrachial dystonic seizures and memory impairment [16,17]. Given the variability in clinical presentations, diagnosing AIE requires specific autoantibody testing to guide treatment decision

Sjögren's Syndrome

Well-known serological markers in Sjögren's syndrome, anti-Ro/SSA and anti-La/SSB antibodies, are mostly linked to exocrine gland dysfunction [18]. Recent studies have shown that these antibodies are also present in the CNS, with evidence of intrathecal synthesis, suggesting that neuroinflammatory involvement can occur in some patients with Sjögren's syndrome [19]. In patients with CNS involvement, antiganglioside M1 antibodies (both IgG and IgM) have been reported, contributing to neurological symptoms by disrupting neuronal membrane integrity and synaptic function [20,21]. Additionally, anti-muscarinic receptor antibodies have been implicated in autonomic nervous system dysfunction, a common feature in Sjögren's syndrome [22]. While these antibodies are not specific to Sjögren's syndrome, their presence in patients with neurological symptoms underscores the multisystem nature of the disease and highlights the importance of considering neuroinflammatory involvement in Sjögren's patients, particularly those presenting with neurological symptoms such as peripheral neuropathy, cognitive dysfunction, or, less commonly, myelitis or encephalitis [20-22].

Systemic Lupus Erythematosus

Systemic lupus erythematosus is a multisystem autoimmune disease characterized by the production of a broad array of autoantibodies, which contribute to the wide range of clinical manifestations seen in the disease [23]. The anti-phospholipid antibody family, which includes anti-cardiolipin antibodies, lupus anticoagulant, and anti-beta-2-glycoprotein antibodies, plays a crucial role in thrombosis and vascular dysfunction, promoting clot formation by targeting proteins involved in the blood clotting cascade [23,24].

In addition to their vascular effects, SLE-specific autoantibodies can also target neuronal tissue, leading to neuropsychiatric symptoms in affected individuals [25]. For instance, anti-ribosomal P antibodies have been linked to psychosis, seizures, and mood disorders in patients with SLE [25]. Moreover, anti-double-stranded DNA (anti-dsDNA) antibodies have been shown to cross-react with NMDA receptors, leading to blood-brain barrier (BBB) disruption, inflammation, and neuropsychiatric manifestations [26,27]. This interaction between autoantibodies and the CNS underscores the complexity of SLE, particularly in the development of neuropsychiatric SLE (NPSLE).

These findings along with their diagnostic utility have been summarized in Table 1.

**Table 1:** Key antibodies specific to each condition, along with their diagnostic utility. IgG: immunoglobulin G; AQP4: aquaporin-4; MOG: myelin oligodendrocyte glycoprotein; MS: multiple sclerosis; CNS: central nervous system; NMDA: N-methyl-D-aspartate; LGI1: leucine-rich glioma-Inactivated 1 protein; Caspr2: contactin-associated protein-like 2; AMPA: alpha-amino-3-hydroxy-5-methyl-4-isoxazolepropionic acid; GABA-B: gamma-aminobutyric acid type B; dsDNA: double-stranded DNA; BBB: blood brain barrier; Anti-Ro/SSA: Anti-Ro/Sjögren's Syndrome A; anti-La/SSB: anti-La/Sjögren's syndrome B.

Condition	Key Antibodies	Diagnostic Utility	Reference
Neuromyelitis Optica	Serum IgG autoantibodies against AQP4 IgG antibodies to MOG (in subset of AQP4-negative cases)	Helps differentiate from MS; antibodies may persist during remission but do not predict long-term disease course or therapy response.	[9,10]
Multiple Sclerosis	CNS oligoclonal IgG bands	Indicate ongoing CNS inflammation; not exclusive to MS	[13,14]
Autoimmune Encephalitis	Antibodies against intracellular, synaptic, and neuronal cell-surface proteins (e.g., NMDA receptor, LGI1, Caspr2, AMPA receptor, GABA-B receptors)	Various clinical presentations depending on targeted antibodies	[16,17]
Sjögren's Syndrome	Anti-Ro/SSA and anti-La/SSB antibodies linked to exocrine gland dysfunction Antibodies targeting gangliosides and muscarinic receptors in nervous system involvement	Association with autonomic dysfunction and other neurological symptoms.	[18,19]
Systemic Lupus Erythematosus	Anti-phospholipid antibodies (e.g., anti-cardiolipin, lupus anticoagulant, anti-beta-2-glycoprotein) Anti-ribosomal P antibodies Anti-NMDA antibodies cross-reacting with dsDNA antibodies	Associated with procoagulant effects, neuropsychiatric symptoms (e.g., psychosis, mood disorders), and potential BBB compromise leading to CNS inflammation.	[24,25]

Clinical Features

Neuromyelitis Optica

Severe bilateral ON is the usual presentation of NMO, which results in significant vision impairment. In NMO, ON frequently presents as abrupt loss of vision and problems with color vision [28]. The most frequent initial sign of optic nerve involvement is decreased visual acuity in the affected eye, accompanied by pain that gets worse with movement. The involvement can be bilateral or unilateral [29]. Within five years of commencement, half of the patients experience functional visual loss [30].

One of the main characteristics of NMO is transverse myelitis, which is characterized by inflammation of the spinal cord. LETM is a condition that frequently results from myelitis in NMO that affects three or more spinal segments [31]. Severe back pain, weakness, sensory abnormalities, and loss of bowel and bladder control are among the symptoms [30]. Like ON, myelitis bouts can be intermittent and relapsing. In addition to neuropathic pruritus and abrupt sensorineural hearing loss, other sensory complaints may also be present [32,33]. Area postrema syndrome is specific to NMO and involves inflammation in the area postrema, a region in the medulla. Hiccups, nausea, and vomiting may ensue from this [34]. NMO may affect various areas of the brainstem in addition to area postrema syndrome, resulting in symptoms such as facial numbness or weakness, double vision, and difficulties with coordination and balance [35]. Severe cases of NMO may involve respiratory muscle weakness due to the involvement of the upper cervical spinal cord, leading to respiratory failure [30]. It can also affect the hypothalamus, resulting in symptoms such as sleep disturbances, temperature dysregulation, and disorders of appetite and thirst [36].

In summary, NMO is characterized by ON, transverse myelitis, and area postrema syndrome, leading to significant visual and neurological impairment. It can affect various regions of the brain and spinal cord, causing relapsing episodes of disability. Understanding these core features is essential for diagnosing and managing NMO effectively.

Multiple Sclerosis

MS is characterized by a variety of neurological symptoms that arise from demyelination and axonal damage in the CNS. Inflammatory processes that impact the myelin sheath of the optic nerve can result in ON, a prevalent early sign [37]. Motor weakness is a reflection of poor signal transmission across motor pathways, whereas sensory problems like paresthesia are caused by altered sensory nerve conduction [38]. Cerebellar involvement-related ataxia is characterized by poor coordination as a result of damaged cerebellar circuitry [39]. Fatigue linked to MS is a multifaceted illness with intricate causes. According to Patejdl et al., there are a few hypothesized explanations, such as immune-mediated inflammation that affects neurotransmitter systems, malfunction of the hypothalamic-pituitary-adrenal axis, and altered mitochondrial activity [40]. Functional neuroimaging studies reveal aberrant activation patterns in brain regions involved in fatigue perception and regulation, such as the prefrontal cortex and basal ganglia [41,42]. 

White matter lesions, gray matter atrophy, and cortical thinning are among the structural alterations in the CNS linked to cognitive deficits in MS [43]. Cognitive impairment is influenced by disturbances in the functional connectivity of large-scale brain networks, including the frontoparietal and default mode networks [44]. MS-related cognitive impairment is also attributed to neuroinflammatory processes, oxidative stress, and synaptic disruption [45,46]. Maladaptive plasticity in the CNS, failure of upper motor neurons, and altered spinal reflexes interact intricately to cause spasticity in MS [47]. According to neurophysiological research, there is a decrease in presynaptic inhibition and an increase in excitability of spinal motor neurons, which results in hyperreflexia, heightened muscular tone, and spasticity [48].

Detrusor hyperactivity, compromised sphincter control, and reduced bladder sensation are the causes of neurogenic bladder dysfunction in MS [49,50]. Studies using neuroimaging show anatomical alterations in brain regions related to bladder control, including the cerebral cortex and the pontine micturition center [51,52]. Constipation and fecal incontinence are hallmarks of neurogenic bowel dysfunction, which is caused by a disruption in the CNS's control over colonic motility and rectal sphincter function [53,54]. A complex combination of biological, psychological, and social factors influences emotional disorders in MS, such as depression, anxiety, and mood swings [55]. Neuroinflammation, changes in neuroendocrine signaling pathways, and dysregulation of monoaminergic neurotransmitter systems are among the neurobiological mechanisms [56,57].

To conclude, MS is a multifactorial autoimmune disease affecting the CNS. It causes ON, motor and sensory dysfunction, cognitive impairment, fatigue, and spasticity. The disease also impacts bladder and bowel control and can lead to emotional disorders. Understanding these diverse symptoms is critical for the comprehensive management of MS.

Autoimmune Encephalitis

Alterations in mental status, from mild confusion to profound encephalopathy with diminished awareness, are frequently associated with AIE [58]. Individuals may display cognitive abnormalities including disorientation, memory loss, and issues with executive function and attention [58,59]. Multiple brain regions may be affected by the inflammatory process, which can result in a variety of symptoms, including movement difficulties, focal neurological deficits (such as weakness or anomalies in sense perception), and seizures [60]. AIE frequently presents with seizures, which can be focal, resulting in loss of consciousness or not, widespread, or refractory status epilepticus [61]. The fundamental processes entail antibody-induced impairment of synaptic proteins, ion channels, or receptors that are essential for preserving neuronal excitability and inhibitory regulation [62].

A number of movement disorders, such as chorea, dystonia, myoclonus, tremors, and ataxia, can be associated with AIE [63]. The malfunction of basal ganglia circuits, cerebellar pathways, or other motor control regions is frequently caused by antibodies, leading to these disorders [64]. Certain forms of AIE present with autonomic symptoms, including dysautonomia. Pupil irregularities, gastrointestinal dysmotility, urine retention or incontinence, and orthostatic hypotension are a few examples of this [65]. These symptoms are caused by dysregulation of the brainstem and hypothalamus's autonomic centers as well as the effects of antibodies on autonomic neurotransmission. 

These deficits arise from disruptions in neuronal networks involved in cognitive processes, caused by autoimmune-mediated injury to certain brain regions. Psychological symptoms are common in cases of AIE and may appear either before or after neurological symptoms. Acute psychosis, delusions, agitation, paranoia, and mood disorders including depression or mania are among the symptoms that patients may exhibit [66]. The psychiatric phenomenology of AIE is attributed to abnormalities in neurotransmitter systems and limbic structures involvement [67].

In conclusion, AIE is an autoimmune disorder that primarily affects the brain, leading to cognitive dysfunction, seizures, movement disorders, and autonomic symptoms. Psychiatric disturbances such as psychosis and mood disorders are also common. The pathophysiology involves antibody-mediated damage to neural circuits, requiring prompt recognition and treatment.

Sjögren's Syndrome

Peripheral neuropathy brought on by Sjögren's syndrome may result in sensory and/or motor deficits in the extremities. Motor neuropathy can cause muscle weakness, cramping, or difficulty with fine motor activities. Sensory neuropathy can manifest as paresthesia, numbness, tingling, or burning sensations. The fundamental causes are interruption of nerve conduction and injury to peripheral nerves caused by the immune system [68]. Nerve conduction studies (NCS) and electromyography (EMG) are useful tools for determining the kind and degree of peripheral nerve involvement.

Numerous cranial neuropathies can result from Sjögren's syndrome's impact on the cranial nerves. For instance, trigeminal neuropathy can result in tingling, numbness, or discomfort in the face [69]. Vision loss or other visual problems may arise from optic neuropathy. Symptoms including diplopia, dysphagia, or dysarthria may result from other cranial nerve involvement [69].

One acknowledged neurological symptom of Sjögren's syndrome is neurocognitive impairment. Individuals may struggle with executive function, memory, focus, attention, and processing speed [70]. Studies using neuroimaging, such as positron emission tomography (PET) and magnetic resonance imaging (MRI), can identify anatomical and functional alterations in the brain linked to cognitive impairment in Sjögren's syndrome [71]. Sjögren's syndrome is frequently accompanied by headaches of various types and intensities. Patients may suffer from headaches of the tension type, migraines, or headaches brought on by inflammation of the sinuses or problems with the cervical spine [72]. Furthermore, variables including dryness, stress, or hormone fluctuations may cause or worsen headaches linked to Sjögren's syndrome.

The neurological symptoms of Sjögren's syndrome could encompass anxiety and despair as well as mood problems. Mood disorders may be caused by exhaustion, chronic pain, cognitive impairment, or the emotional toll of having a long-term autoimmune disease [73]. Mood regulation may also be influenced by changes in the function of the hypothalamic-pituitary-adrenal axis, neuroinflammatory processes, and neurotransmitter imbalances [74].

To summarize, Sjögren's syndrome can lead to peripheral and cranial neuropathy, cognitive dysfunction, headaches, and mood disorders. The complex interplay of autoimmune mechanisms and neuroinflammation affects various parts of the nervous system. Effective management includes addressing both neurological and emotional symptoms.

Systemic Lupus Erythematosus

Neuropsychiatric systemic lupus erythematosus is the collective term for a range of symptoms associated with neurological involvement in SLE. Mood problems (anxiety, sadness), focal neurological deficits, psychosis, seizures, headaches, and cognitive dysfunction (memory impairment, poor attention, and executive dysfunction) are typical hallmarks [75,76]. A higher incidence of cerebrovascular events, such as cerebral venous thrombosis (CVT), transient ischemic episodes (TIAs), and ischemic strokes, is linked to SLE [77]. Numerous factors, such as antiphospholipid antibodies, vasculitis, and accelerated atherosclerosis in the context of lupus-related inflammation and vascular damage, might cause these occurrences [77].

Patients with SLE are not immune to seizures, although focal seizures are more prevalent than generalized ones [7]. Numerous elements are involved in the underlying mechanisms, such as lupus cerebritis (inflammatory brain involvement), cortical or subcortical abnormalities, and antiphospholipid-associated thrombosis [75]. Convulsions may manifest as a presenting characteristic or happen when SLE is being treated [7]. Numerous interrelated elements play a complex role in the pathophysiology, including neuroinflammation, disruption of the BBB, neuronal injury, and effects of autoantibodies on synapse function [78]. Peripheral neuropathy brought on by SLE can result in deficiencies in motor, sensory, or mixed nerve function. In patients with SLE, peripheral neuropathy may be caused by direct antibody-mediated nerve injury, immune complex deposition, or vasculitis that affects the peripheral nerves [79]. Clinical manifestations may include reduced reflexes, neuropathic discomfort, muscular weakness, and sensory abnormalities.

Myelitis or transverse myelitis, which is characterized by inflammation and damage to the tissues of the spinal cord, can result from spinal cord involvement in SLE [80]. Depending on the degree of spinal cord involvement, this can cause sensory deficiencies, motor weakness, bowel and bladder problems, and occasionally ascending or descending paralysis [81]. SLE can cause uveitis, optic neuropathy, retinopathy, and other ocular symptoms by affecting the optic nerves and eyes [82]. Inflammation of the optic nerve is the hallmark of optic neuritis, which can result in pain while moving the eyes and potentially vision loss [7].

In patients with SLE, intracranial hypertension (resulting from medication or lupus cerebritis), thrombosis associated with antiphospholipid antibodies, and tension-type headaches are among the many possible causes of headaches [83]. Psychosis (delusions, hallucinations), mood disorders (depression, anxiety), and personality changes are common psychiatric symptoms of SLE [84]. The etiology of psychiatric symptoms in SLE is complex and includes immune-mediated inflammation, modulation of neurotransmitters, drug side effects, and psychosocial variables [85].

In conclusion, SLE’s neuropsychiatric manifestations include mood disorders, seizures, cognitive dysfunction, and cerebrovascular events. Peripheral neuropathy, myelitis, and optic neuropathy are common neurological features. The complexity of the disease requires comprehensive management to address both the neurological and psychiatric aspects.

Overlap between the diseases

Neuromyelitis Optica and Multiple Sclerosis

There may be clinical and radiological similarities between MS and NMO. Differentiating between the two disorders at first can be difficult because they can both manifest as optic neuritis, myelitis, and brain lesions [86]. Nonetheless, a few characteristics help to differentiate them: While MS usually appears with shorter lesions and more frequent brain involvement, NMO frequently shows bilateral optic nerve involvement, LETM, and a preference for attacks in the central gray matter of the spinal cord [87]. Improved lesion characterization and localization have been made possible by advanced neuroimaging techniques, including T2-weighted imaging and MRI with particular sequences like fluid-attenuated inversion recovery (FLAIR), which help with differential diagnosis [88]. Additionally, the discovery of specific autoantibodies such as AQP4-IgG and MOG-IgG, has revolutionized the accuracy of diagnosis, with AQP4-IgG being highly specific for NMO [86].

To conclude, while MS and NMO share several overlapping features, distinct differences in lesion localization, imaging findings, and autoantibody presence allow for accurate differentiation between the two conditions.

Neuromyelitis Optica and Autoimmune Encephalitis

Due to their similar neurological symptoms and possible autoimmune causes, NMO and AIE are related. Akin to AIE, patients with NMO may exhibit neuropsychiatric symptoms like cognitive impairment, seizures, and behavioral abnormalities [89]. It takes a multifaceted approach to distinguish between these conditions, including a thorough clinical evaluation, neuroimaging studies (MRI, PET scans), CSF analysis for inflammatory markers, and antibody testing to pinpoint particular subtypes of AIE or NMO spectrum disorders [90]. Specific antibodies associated with AIE, such as anti-NMDAR or anti-LGI1, are not typically found in NMO, and the presence of AQP4-IgG or MOG-IgG favors a diagnosis of NMO spectrum disorder. While both conditions can be immune-mediated, they target different antigens and CNS regions [16,17].

To summarize, while the neuropsychiatric symptoms of NMO and AIE may overlap, they can be differentiated through clinical evaluations, advanced neuroimaging, and targeted antibody testing.

Neuromyelitis Optica and Sjögren's Syndrome

It has been established that NMO and Sjögren's syndrome coexist or share characteristics, despite their relative rarity, especially when it comes to neurological symptoms, resulting in neuropathic pain, sensory neuropathy, or cognitive impairment [67]. Potential autoimmune processes linking both conditions may be indicated by the presence of anti-Sjögren's Syndrome A (SSA)/Ro antibodies in patients with NMO [91]. Myelitis can be a neurological symptom of either disorder; however, LETM is more typical in NMO, whereas Sjögren's syndrome-associated myelitis typically presents with a more variable clinical picture [85]. Accurate diagnosis and focused treatment approaches depend on advanced immunological profiling, which includes cytokine analysis and antibody testing [92].

In conclusion, NMO and Sjögren's syndrome may present with overlapping neurological features, but differences in myelitis presentation and specific autoantibody profiles help in distinguishing the two conditions.

Neuromyelitis Optica and Systemic Lupus Erythematosus

The overlap between NMO and SLE extends to their shared neuropsychiatric manifestations and potential neurological involvement. SLE can affect the central and peripheral nervous systems, leading to a variety of neurological symptoms, including seizures, cognitive dysfunction, and myelopathy [75]. Accurate diagnosis and treatment planning depend on neuroimaging modalities like MRI, especially with spinal cord sequences, and antibody testing for anti-double-stranded DNA antibodies in SLE or AQP4-IgG in NMO [25,85]. Additionally, a subgroup of SLE patients with neurological manifestations has been found to have NMO-IgG (anti-AQP4 antibodies) in recent investigations, suggesting that these autoimmune processes may overlap or coexist [93].

To summarize, while NMO and SLE share certain neurological manifestations, distinct autoimmune markers, and neuroimaging findings are crucial for accurate diagnosis and management of these disorders.

In summary, while notable overlaps exist among the clinical presentations of NMO, MS, AIE, Sjögren's syndrome, and SLE, recognizing the distinct characteristics, diagnostic markers, and treatment pathways for each condition is essential. Table 2 highlights the diverse neurological symptoms and manifestations associated with each condition, along with the overlapping features between the various conditions.

**Table 2:** Neurological manifestations along with the overlapping features between the various conditions ON: optic neuritis; LETM: longitudinally extensive transverse myelitis; MS: multiple sclerosis; SLE: systemic lupus erythematosus; NMO: neuromyelitis optica; AIE: autoimmune encephalitis

Condition	Common Clinical Features	Overlapping Features	Reference
Neuromyelitis Optica	ON, LETM, nausea/vomiting	Myelitis (seen in MS, Sjögren’s, SLE)	[28,30]
Multiple Sclerosis	Fatigue, cognitive decline, ataxia, ON	ON (shared with NMO)	[37,38]
Autoimmune Encephalitis	Seizures, psychiatric symptoms, autonomic dysfunction	Cognitive impairment (overlaps with MS, SLE)	[58,59]
Sjögren's Syndrome	Peripheral neuropathy, cranial neuropathies	Neuropathy (overlaps with NMO, SLE)	[68,69]
Systemic Lupus Erythematosus	Mood disorders, seizures, psychosis	Cognitive deficits (shared with AIE, MS)	[75,76]

Treatment approaches

Neuromyelitis Optica

Immunosuppressive treatment, which targets AQP4 in the CNS, remains the cornerstone of managing NMO. For acute relapses, high-dose methylprednisolone, a corticosteroid, is frequently employed to reduce inflammation and rapidly stabilize disease activity [94]. This intervention is often the first line of treatment during flare-ups. However, the long-term use of corticosteroids like methylprednisolone is limited due to the potential for significant side effects, including osteoporosis, weight gain, and diabetes [94]. As a result, other immunosuppressive medications are used for long-term care and relapse prevention [95]. Rituximab, a monoclonal antibody that targets CD20-positive B cells, has shown promise in slowing disability progression and relapse rates in NMO patients [95,96]. Other immunosuppressants, including azathioprine, mycophenolate mofetil, and methotrexate, are utilized as steroid-sparing agents or in combination therapy to help maintain disease stability and reduce relapse frequency [96]. In cases of acute episodes or resistant patients, intravenous immunoglobulin (IVIG) or plasma exchange (PLEX) may be explored. New treatments targeting the complement system, including eculizumab, have demonstrated promise in the treatment of NMO [97,98].

Multiple Sclerosis

The cornerstone of MS treatment is disease-modifying therapies (DMTs), which aim to decrease the frequency of relapses, delay disability progression, and maintain neurologic function [99]. A variety of DMTs are available, such as monoclonal antibodies targeting immune cells or pathways, glatiramer acetate, interferons, and oral medications like fingolimod, dimethyl fumarate, and teriflunomide [99]. The selection of these therapies depends on the severity, phenotype, and unique patient characteristics. Monoclonal antibodies have revolutionized MS treatment by providing highly targeted immunomodulation [99]. An α4-integrin inhibitor called natalizumab lowers immune cell migration into the CNS, which reduces inflammatory activity [99,100]. Alemtuzumab targets CD52-positive lymphocytes, leading to immune reconstitution and modulation [100]. Crelizumab and cladribine are recent additions to the MS therapy arsenal, showing promise in treating active secondary progressive MS and relapsing-remitting MS [100]. These agents offer additional therapeutic options for patients with varying disease courses and treatment responses. In addition to DMTs, methylprednisolone remains a standard treatment for acute relapses in MS, as it rapidly reduces inflammation and protects the CNS from further damage [100]. Symptomatic management plays a crucial role in addressing MS-related symptoms, including spasticity, fatigue, pain, and cognitive impairment. Rehabilitation programs, physical therapy, and symptomatic medications are integral components of comprehensive MS care [101].

Autoimmune Encephalitis

Supportive care, targeted immunotherapy, and immunosuppression are used in the treatment of AIE [102]. Methylprednisolone and other high-dose corticosteroids are commonly used as first-line treatments to lower autoantibody levels and reduce inflammation [102]. This corticosteroid therapy is pivotal in rapidly managing acute inflammation in AIE cases. Another essential component of treatment is IVIG, which helps modulate the immune system through passive immunotherapy [103]. PLEX may be used to eliminate circulating autoantibodies and inflammatory mediators in severe or refractory cases [104]. Rituximab is frequently used as a maintenance therapy or second-line medication to prevent relapses in AIE [105]. For targeted immunotherapy to be effective, it is crucial to identify the precise neuronal surface antibodies implicated in the disease, as different autoantibodies may require distinct treatment strategies. Long-term management of AIE typically involves close observation, immunosuppressive maintenance therapy, and supportive care to minimize neurological sequelae and optimize outcomes.

Sjögren's Syndrome

The treatment of Sjögren's syndrome aims to alleviate symptoms, prevent complications, and manage systemic manifestations. Hydroxychloroquine is frequently used to treat the systemic symptoms of Sjögren's syndrome and reduce disease activity [106]. In cases of refractory symptoms or widespread systemic involvement, immunosuppressive treatments such as methotrexate, azathioprine, or mycophenolate mofetil may be considered to reduce inflammation and prevent organ damage [107]. Biologic agents targeting B cells (e.g., rituximab) or cytokines (e.g., tumor necrosis factor (TNF)-alpha inhibitors) are reserved for severe or refractory cases, underscoring the importance of a stepwise approach to treatment escalation [108]. To alleviate moisture-related symptoms such as dry mouth and dry eyes (sicca symptoms), topical medications, artificial tears, and saliva replacements are recommended [109]. Nonsteroidal anti-inflammatory drugs (NSAIDs) or low-dose corticosteroids may be used for systemic signs such as arthralgia, myalgia, and fatigue [109].

Systemic Lupus Erythematosus

The primary goals of managing SLE are to control disease activity, prevent flare-ups, manage organ involvement, and maximize quality of life [110]. Hydroxychloroquine, a mainstay of SLE treatment, has immunomodulatory effects and reduces disease activity, especially for articular and cutaneous symptoms [110]. Methylprednisolone is commonly used for managing acute flares and severe symptoms, with glucocorticoids being tapered to the lowest effective dose over time to reduce long-term side effects [111]. For maintenance therapy and steroid-sparing, disease-modifying medications such as methotrexate, azathioprine, and mycophenolate mofetil are used [111]. In refractory cases of lupus nephritis, biologic treatments that target certain pathways in SLE, such as B cell depletion with rituximab or suppression of cytokines like IL-6 (e.g., tocilizumab) or B cell activating factor (BAFF) (e.g., belimumab), have demonstrated success [112]. Individualized treatment plans, close monitoring, and multidisciplinary care are essential for optimal SLE management.

Table 3 provides a comprehensive summary of treatment strategies for each autoimmune disorder, outlining immunosuppressive approaches, acute relapse management, and long-term therapeutic options. The table highlights the role of monoclonal antibodies, corticosteroids, and emerging biologics in managing these conditions, aiding in individualized patient care.

**Table 3:** Treatment approaches for each autoimmune disorder AQP4: aquaporin-4; IVIG: intravenous immunoglobulin; PLEX: plasma exchange; DMTs: disease modifying therapies; TNF: tumor necrosis factor; NSAIDs: nonsteroidal anti-inflammatory drugs

Disease	Treatment Approaches	Reference
Neuromyelitis Optica	Immunosuppressive Treatment: Targets AQP4 with medications like rituximab, azathioprine, mycophenolate mofetil	[94,95]
	Acute Relapses: High-dose corticosteroids (methylprednisolone), IVIG, PLEX	
	Long-term Management: Rituximab, azathioprine, mycophenolate mofetil, methotrexate	
	Resistant Cases: IVIG, PLEX, eculizumab	
Multiple Sclerosis	DMTs: Monoclonal antibodies (natalizumab, alemtuzumab), interferons, oral medications (fingolimod, dimethyl fumarate)	[99,100]
	Symptomatic Management: Address spasticity, fatigue, pain, cognitive impairment	
Autoimmune Encephalitis	First-line: Methylprednisolone	[102,103]
	For severe cases: IVIG and PLEX	
	Maintenance: Rituximab, tailored immunosuppression based on specific neuronal surface antibodies	
	Supportive Care: Long-term immunosuppressive maintenance therapy, close observation.	
Sjögren's Syndrome	Systemic Symptoms: Hydroxychloroquine	[106,107]
	Refractory Cases: Methotrexate, azathioprine, mycophenolate mofetil	
	Severe Manifestations: Biologics targeting B cells (e.g., rituximab) or cytokines (e.g., TNF-alpha inhibitors)	
	Symptomatic Relief: Topical treatments, NSAIDs, low-dose corticosteroids.	
Systemic Lupus Erythematosus	Mainstay for disease activity: Hydroxychloroquine	[110,111]
	Acute Flares: Glucocorticoids (prednisone)	
	Maintenance: Methotrexate, azathioprine, mycophenolate mofetil	
	Severe Cases: Biologics (rituximab, tocilizumab, belimumab) for nephritis or refractory symptoms	

**Figure 2:**
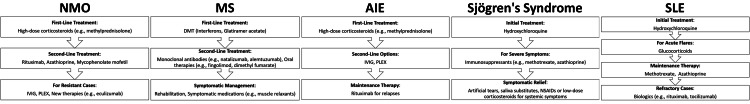
Treatment Approaches for Autoimmune CNS Diseases NMO: neuromyelitis optica; IVIG: IV immunoglobulins; PLEX: plasma exchange; MS: multiple sclerosis; DMT: disease modifying therapies; AIE: autoimmune encephalitis; NSAIDs: non-steroidal anti inflammatory drugs; SLE: systemic lupus erythematosus Image credits: Tabish Siddiqui

Figure 2 summarizes the various therapies available to manage these autoimmune CNS diseases.

Advancements in diagnostic testing, emerging therapies, and novel drug targets

Advancements in Diagnostic Testing

Recent technological advancements have significantly improved the diagnostic capabilities for autoimmune CNS diseases. High-resolution MRI with specialized sequences, such as FLAIR, allows for precise visualization of CNS lesions, which is critical for the early detection and characterization of disease activity [113]. Techniques like diffusion tensor imaging (DTI) and functional MRI provide detailed insights into tissue microstructure and brain connectivity, which are valuable for assessing therapy responses and tracking disease progression [113]. These imaging modalities are complemented by the development of novel biomarkers, which enhance disease classification and facilitate personalized treatment strategies [114].

The identification of specific autoantibodies has transformed the diagnostic approach for autoimmune CNS diseases. For example, the detection of anti-AQP4 in NMO and anti-MOG in demyelinating diseases allows for accurate diagnosis and guides therapeutic decisions [94]. In addition, biomarkers like neurofilament light chain and S100 proteins offer insights into disease activity and prognosis, aiding in the management of these conditions [113]. Furthermore, next-generation sequencing (NGS) technologies have advanced genomic profiling, enabling the identification of genetic predispositions and potential therapeutic targets for autoimmune CNS diseases [114]. This molecular approach allows for more tailored and effective treatment plans based on each patient’s genetic and immunological profile [114].

Emerging technologies, such as proteomics, metabolomics, and transcriptomics, provide comprehensive molecular insights that contribute to a deeper understanding of disease mechanisms [114]. These "omics" approaches facilitate the identification of disease-specific biomarkers, improving early diagnosis and enabling more accurate classification of disease subtypes [113]. The integration of these technologies holds promise for personalized treatment strategies, offering a more precise approach to patient care [113].

Emerging Therapies

Biological therapies, particularly monoclonal antibodies, have become central to the treatment of autoimmune CNS diseases. These therapies target specific immune cell populations or cytokines critical to disease pathophysiology, offering more effective disease management [114]. Monoclonal antibodies such as rituximab have been shown to reduce relapse rates and slow disease progression in conditions like NMO and MS by modulating B-cell activity [95,96]. Rituximab and other B-cell-depleting therapies restore immune balance, reducing the frequency of relapses and improving patient outcomes [95]. Complement inhibitors like eculizumab have shown promise, particularly in conditions like NMO, where complement-mediated damage plays a central role [97]. As emerging therapies, these approaches offer the potential for long-term remission and more effective disease control in autoimmune CNS conditions [114].

Novel Drug Targets

Advancements in molecular diagnostics have led to the identification of specific autoantibodies, which are crucial for enhancing diagnostic accuracy and informing targeted therapeutic approaches [95]. For instance, autoantibodies such as anti-AQP4 in NMO and anti-MOG in demyelinating diseases allow for a more precise classification of these diseases and enable personalized treatment [94]. Targeted therapies based on these biomarkers are increasingly integral to disease management, offering clinicians the ability to tailor interventions to the patient’s individual immunological profile [95]. The ongoing discovery of new biomarkers, coupled with innovative technologies, is shaping the future of personalized medicine in autoimmune CNS diseases.

Emerging diagnostic technologies face challenges in standardization and high costs. Monoclonal antibodies and biologic therapies are limited by side effects, safety concerns, and expense. Novel drug targets require further clinical trials and pose risks like resistance and incomplete disease understanding.

Existing knowledge gaps and potential avenues for future research

Despite advancements, many gaps remain in our understanding of autoimmune diseases, particularly in the interplay of genetic, environmental, and immunological factors. Research should focus on the following areas.

Genetic and Environmental Interactions

Autoimmune diseases involve complex interactions between genetic predispositions and environmental triggers. Human leukocyte antigen (HLA) alleles, such as HLA-DRB1*15:01 for MS, and factors like Epstein-Barr virus have been identified as contributing factors [115,116]. Further research should explore how genetic and environmental factors interact at the molecular level to trigger disease, with a focus on gene-environment interactions and epigenetic modifications [115].

Biomarkers for Early Detection and Prognostication

Current biomarkers are insufficient for early diagnosis and predicting disease progression. For instance, anti-dsDNA antibodies, while useful for lupus, lack sensitivity for early-stage detection [26]. Future research should focus on novel biomarkers, including autoantibodies and microRNAs, for early detection and predicting treatment responses [117]. Studies on biomarkers like neurofilament light chain in MS show promise for prognostication [117].

Personalized Medicine and Treatment Optimization

Personalized medicine holds great promise but faces challenges like variability in patient responses and high therapy costs [118]. Research should develop biomarker-guided therapy algorithms and comparative efficacy studies, such as those assessing DMTs in MS [118]. Machine learning and Artificial Intelligence (AI) approaches can help optimize treatment decisions by integrating genetic, clinical, and environmental data.

Neuroimmunological Mechanisms and CNS Involvement

The mechanisms of CNS involvement in autoimmune diseases like MS and NMO remain poorly understood. Research should investigate the role of cytokines (e.g., TNF-alpha, IL-1 beta) in neuroinflammation and explore neuroprotective strategies like mesenchymal stem cells for remyelination [99].

Identifying Risk Factors for Disease Onset and Progression

Identifying risk factors for disease onset and progression, such as smoking and vitamin D deficiency, is crucial [119]. Large cohort studies are needed to uncover how these factors contribute to autoimmune diseases. Early identification of high-risk individuals can lead to targeted prevention strategies and more effective management.

Challenges in Integrating Genetic and Environmental Factors into Treatment Algorithms

Integrating genetic and environmental factors into treatment algorithms is challenging due to complex gene-environment interactions [118]. Future research should develop adaptive algorithms that consider these factors to guide personalized treatments, addressing ethical concerns regarding genetic privacy and discrimination.

Limitations

While efforts have been made to encompass a wide range of autoimmune disorders, such as NMO, MS, AIE, Sjögren's syndrome, and SLE, the review's scope may still overlook less well-researched areas or recent developments. This limitation could affect the comprehensiveness of the review's findings and its relevance to current clinical practice. While efforts have been made to provide a balanced synthesis, inherent biases in interpreting data and selecting studies may affect the objectivity of conclusions drawn regarding the complex interactions and treatment approaches discussed.

## Conclusions

The comparison of NMO with other autoimmune diseases such as MS, AIE, Sjögren's syndrome, and SLE reveals both distinct and shared pathophysiological features, emphasizing the complexity of these disorders. Each disease targets the CNS in unique ways, with NMO characterized by ON and LETM driven by AQP4 autoantibodies, contrasting with MS's widespread demyelination and persistent oligoclonal bands in its diagnosis. AIE involves autoimmune attacks against synaptic proteins, while Sjögren’s syndrome and SLE exhibit multisystem involvement, affecting both exocrine glands and neural tissues. Despite these differences, there is notable overlap, such as shared neurological symptoms like myelitis between NMO and AIE, or peripheral neuropathy between NMO and Sjögren's syndrome. The varying antibody profiles - AQP4 and MOG antibodies in NMO, oligoclonal bands in MS, and neuronal antibodies in AIE - further underscore the diagnostic challenges posed by these diseases.

To effectively bridge existing knowledge gaps and optimize patient outcomes, future research must prioritize interdisciplinary approaches that integrate genetic, environmental, and immunological data. Refining diagnostic criteria, improving early detection strategies, and understanding how genetic predispositions and environmental triggers contribute to disease progression are crucial. Personalized treatment algorithms, driven by biomarker-guided therapies and comparative efficacy studies, hold great promise for improving patient responses while minimizing side effects. Moreover, neuroprotective strategies and deeper insights into the neuroimmunological mechanisms underlying CNS involvement are essential for developing targeted immunotherapies. A concerted effort from researchers and clinicians is needed to translate these advancements into precise diagnoses, tailored treatments, and ultimately, improved quality of life for individuals affected by these complex autoimmune CNS disorders.
